# Predictors of Health-Related Quality of Life among Healthcare Workers in the Context of Health System Strengthening in Kenya

**DOI:** 10.3390/healthcare9010018

**Published:** 2020-12-25

**Authors:** Rose Nabi Deborah Karimi Muthuri, Flavia Senkubuge, Charles Hongoro

**Affiliations:** 1 School of Health Systems and Public Health (SHSPH), Faculty of Health Sciences, University of Pretoria, Pretoria 0028, Gauteng Province, South Africa; flavia.senkubuge@up.ac.za (F.S.); chongoro@hsrc.ac.za (C.H.); 2 Developmental, Capable and Ethical State Division, Human Sciences Research Council (HSRC), Pretoria 0001, Gauteng Province, South Africa; 3 Faculty of Science, Tshwane University of Technology, Pretoria 0183, Gauteng Province, South Africa; 4 Faculty of Health Sciences, Fort Hare University, Alice 5700, Eastern Cape, South Africa

**Keywords:** health-related quality of life, health measurement, work environment, healthcare workers, health systems

## Abstract

Kenya is among the countries with an acute shortage of skilled health workers. There have been recurrent health worker strikes in Kenya due to several issues, some of which directly or indirectly affect their health. The purpose of this study was to investigate the predictors of health-related quality of life (HRQOL) among healthcare workers in public and mission hospitals in Meru County, Kenya. A cross-sectional study design was undertaken among 553 healthcare workers across 24 hospitals in Meru County. The participants completed the EuroQol-five dimension-five level (EQ-5D-5L) instrument, which measures health status across five dimensions and the overall self-assessment of health status on a visual analogue scale (EQ-VAS). Approximately 66.55% of the healthcare workers reported no problems (i.e., 11,111) across the five dimensions. The six predictors of HRQOL among the healthcare workers were hospital ownership (*p* < 0.05), age (*p* < 0.05), income (*p* < 0.01), availability of water for handwashing (*p* < 0.05), presence of risk in using a toilet facility (*p* < 0.05), and overall safety of hospital work environment (*p* < 0.05). Personal, job-related attributes and work environment characteristics are significant predictors of healthcare workers HRQOL. Thus, these factors ought to be considered by health policymakers and managers when developing and implementing policies and programs aimed at promoting HRQOL among healthcare workers.

## 1. Introduction

In 2014, the United Nations (UN) General Assembly consisting of 193 Heads of State, universally adopted resolution A/RES/70/1 on ‘Transforming our world: the 2030 Agenda for Sustainable Development’ that envisions “A world with equitable and universal access … to health care and social protection, where physical, mental and social well-being are assured” (p. 3) [1]. The resolution contains 17 Sustainable Development Goals (SDGs) and 169 targets. SDG 3 emphasizes healthy lives for all persons of all ages [1]. The sixty-ninth World Health Assembly (WHA) stated that SDG 3, and other health development agendas, could not be achieved without investing in and improving the health workforce [2]. According to a World Health Organization (WHO) report titled, “A universal truth: no health without a workforce” [3], it is paramount to put the health workforce at the center of health policy discourse aimed at strengthening health systems, improving public health outcomes, and achieving health development agendas [3]. The health workforce plays a pivotal role in the achievement of global health agendas such as Universal Health Coverage and SDG 3 by 2030 [3]. However, one of the most significant challenges in the Kenyan health system is a critical shortage of skilled healthcare workers [4].

The WHO recommended minimum threshold of skilled health workers is 2.3 per 1000 population [4]. By 2006, the skilled health workers’ density in Kenya was 1.8 per 1000 [4]. A decline was reported in 2018, when the density of skilled health workers was 1.74 per 1000 population (an equivalent of 17.4 per 10,000 population) [5]. In addition to the acute shortage of skilled health workers [6], the health workforce is also facing neglect in health systems development [7]. In Kenya, the “brain drain” [8], migration [9], poor working conditions [10], poor human resources for health management [11,12], low salary, and delayed payment (or non-payment) of salaries [13], are some of the many challenges which the health workforce encounter, which leads to recurrent health worker strikes [14,15]. In 2017, Kenya experienced two countrywide 100-day doctors strikes, and 150-day nurses strikes [13], which also contributed to low health worker retention. Such strikes adversely impact the quality of healthcare [13] and threaten national and devolved health systems development [16].

In 2016, the WHO report titled ‘Global Strategy on human resources for health: Workforce 2030′ was published [17], following the adoption of related resolution WHA67.24 in 2014 [18]. One of the principles of this global strategy is to “Uphold the personal, employment and professional rights of all health workers, including a safe and decent working environment…” (p. 8) [17]. According to the Constitution of Kenya Article 43 (1) (a): “Every person has a right to the highest attainable standard of health, which includes the right to healthcare services, including reproductive health care.” (p. 31) [19]. This signifies that health is a vital component for every individual (including a health worker), as recognized at the organizational, national, and global levels. Researchers and specialists across various disciplines have a unifying belief that health is vital in healthcare service delivery [20], health systems strengthening [21], and overall human development.

The Constitution of the World Health Organization defines health as, “the complete state of physical, mental and social well-being, not merely the absence of disease or infirmity” (p. 1) [22]. In this study, we assess the health-related quality of life (HRQOL). Currently, there is no universal definition of HRQOL [23]. In this study, HRQOL is defined as a multifaceted concept that delves into the assessment of ones’ self-perceived health status using a multidimensional classification system [24]. Therefore, HRQOL is a value attributed to life, specifically focusing on health-related functional ability (or inability) and perceptions at an individualistic contextual realm [25]. HRQOL has been assessed among healthcare workers in various countries such as China [26], Pakistan [27], South Africa [28], and Greece [29], among others.

In Kenya, studies on HRQOL have been conducted among various populations such as children in Schistosoma haematobium-endemic areas [30], people living with irrigation schemes [31], women living in informal settlements [32], patients undergoing antiretroviral treatment [33,34,35], patients on maintenance hemodialysis [36] and patients who have undergone cataract surgery [37]. However, a study on HRQOL among healthcare workers in Kenya is yet to be conducted. Therefore, the present study contributes to bridging the existing knowledge gap in the public and private-not-for-profit (mission) hospitals. This study aims to investigate the predictors of HRQOL among healthcare workers in public and mission hospitals, Meru County, Kenya. The three research questions that this study aims to answer are:

1. What is the average overall self-assessed health status of the healthcare workers?
2. Is there a statistically significant difference between healthcare workers’ overall self-assessed health status and hospital ownership?
3. What is the statistically significant relationship between healthcare workers’ overall self-assessed health status and personal, job-related, and work environment characteristics?

This study will contribute to the existing literature that policymakers can use to inform the development of an evidence-based health workforce policy. It will also contribute to raising the awareness among policymakers and health development partners on the pivotal role of healthcare workers’ HRQOL in health workforce strengthening, development of resilient health systems, and achievement of the SDG3 target 3.8 on Universal Health Coverage (and indeed attainment of all the remaining 12 targets).

## 2. Materials and Methods

### 2.1. Study Design

A cross-sectional study design was used to investigate HRQOL among healthcare workers in public and mission hospitals in the Meru County of Kenya.

The study was conducted between 15 June and 30 July 2020, which was during the Coronavirus Disease 2019 (COVID-19) pandemic. However, at that time, the COVID-19 cases were relatively few in Meru County. By the end of July, a total of 32 cases had been recorded [38]. Therefore, the COVID-19 pandemic did not adversely impact the data collection process. The healthcare workers were highly cooperative during the data collection phase of this study. However, despite there being low numbers of cases in Meru County at the time of data collection, it is important to note that globally, the COVID-19 pandemic shocked health systems and resulted in healthcare workers experiencing psychological distress and psychosomatic symptoms [39]. For example, in China and Singapore, a narrative review revealed that the COVID-19 global pandemic resulted in healthcare workers experiencing enormous stress especially during spikes of cases which were experienced at different periods across different countries worldwide [39].

### 2.2. Study Setting

Meru County is one of the forty-seven counties in Kenya. The total population by July 2020, was approximately 1,545,714 people [38]. It is primarily a rural area located on the eastern slopes of Mount Kenya and is known for livestock rearing and agriculture, specifically, cash crop and food crop farming [40]. By 2019, there were 183 health facilities across the entire healthcare referral system in Meru County. This study focused on the sub-county and county level, public and mission hospitals (*n* = 24). In Kenya, the majority of hospitals are categorized according to hospital ownership. In the rural areas, hospital ownership is primarily public and mission. This means that public hospitals are owned and operated by the government. In comparison, mission hospitals are owned and operated by private not-for-profit religious organizations.

### 2.3. Study Population and Sample

In Meru County, the total number of human resources for health (HRH) in public and mission sub-county and county hospitals was 1872 by 2019. The present study focused on healthcare workers, also known as healthcare professionals. Healthcare workers, in this study, are individuals who have been trained in the medical field to apply evidence-based medical procedures and principles, geared towards achieving quality healthcare delivery [41]. Our focus was on medically trained healthcare workers, excluding auxiliary staff. Thus, the total number of healthcare workers eligible to participate was 954. The sample size (ss) was calculated using the following formula [42]:

(1) $$\mathrm{ss}=(\frac{Z^{2*\;}\left(p\right)*\left(1-p\right)}{C^{2}})$$

Based on the formula, the total sample size of 566 was determined by the following parameters: the population was 954, Z = Z value at 99% confidence level, C = confidence interval of 3.46, and p = response distribution percentage of 50%.

Using simple random sampling a total of *n* = 566 healthcare workers were selected across the all the public and mission (*n* = 24) hospitals to participate in this study. The health professional cadres presented in this study were doctors, clinical officers, nursing personnel, dentistry personnel, pharmaceutical personnel, medical laboratory scientists, nutritionists, public health specialists, mental health specialists, physiotherapists, radiologists, and health records officers.

### 2.4. Data Collection

#### 2.4.1. Sample Characteristics

Sample characteristics were collected in the socio-demographic section of the instrument. The personal and job-related attributes, and work environment characteristics, constituted the independent variables in this study. The personal and job-related attribute data obtained were in the following categories: hospital ownership, health professional cadre, age, marital status, gender, household size, education attained, years of professional experience, hours worked in a week, in-service training, staff housing, and type of employment. Work environment characteristics data on the healthcare workers’ perception of their working environment related to hygiene, water, sanitation, and occupational hazards in the hospital, were also obtained.

#### 2.4.2. The EQ-5D-5L

The EuroQol-five dimension-five level instrument (EQ-5D-5L) developed by the EuroQol Research Foundation [43] was used to measure HRQOL among healthcare workers. By 2019, the EQ-5D-5L had been translated into more than 180 languages and applied globally [43]. The EQ-5D-5L assessed the respondents’ self-perceived health across five dimensions, namely: mobility, self-care, usual activities, pain/discomfort and, anxiety/depression [43]. Each respondent indicated what they felt across the five dimensions, depending on the boxes ticked, and a five-digit number, e.g., 11,111 (denoting full health), was generated for analytical purposes as per the EuroQol User Guide [43]. The last question in the instrument is the EQ-VAS (EuroQol Visual Analogue Scale) which required the respondents to assess their overall health status on a scale 0–100, where 0 signifies the worst health one can imagine, and 100 signifies the best health the respondent can envisage [43]. The EQ-5D-5L instrument was used after obtaining permission to use the Kenyan version for this study as instructed by the EuroQol Research Foundation. The research team, consisting of the principle investigator and two research assistants, explained the study both in written form (informed consent form) and verbally. After signing the informed consent form, respondents were given the self-complete paper version of the questionnaire. The respondents were informed that they could ask the research team any questions regarding the study, and they completed the questionnaire anonymously. Upon completing the questionnaire, the respondents would return it to the research team. On average, each respondent completed the questionnaire within 10 min.

Pretesting of the data collection instrument was performed among healthcare workers to evaluate its contextual validity and lucidity. The section of the personal, job-related and work environment characteristics was modified to enhance the contextual applicability in our setting.

#### 2.4.3. Statistical Analysis

Data entry was performed in Excel (Microsoft, Washington, DC, USA) and exported to STATA 15.1^®^ (StataCorp., College Station, TX, USA). Analysis of the EQ-5D-5L self-complete paper version was conducted according to the EuroQol User Guide [43]. From the respondents scores across the five dimensions, the EQ-5D-5L health profiles were obtained. From this, the EQ-5D index values were calculated using the EQ-5D-5L Crosswalk Index Value calculator for Windows [43]. After obtaining the index values, measures of central tendency (including the median and interquartile range) were estimated using STATA 15.1^®^ [43]. Using the EQ-VAS as the dependent variable (i.e., the self-reported overall health status score), analysis of variance (ANOVA) and linear multivariate regression analysis was performed using STATA 15.1^®^. The linear multivariate regression model estimated was [44]:

(2) $$Y_{k}=\beta_{0}+\beta_{1}X_{1k}+\beta_{2}X_{2k}+\ldots +\beta_{25}X_{25k}+\in_{k}$$

where $\beta_{0}$ indicates the constant or intercept term capturing the unexplained variations in the dependent variable Y (i.e., EQ-VAS), $\beta_{1}$ indicates the slope coefficient measuring the amount by which Y will change when X changes by a single unit, *k* ranges from 1 to *n*, in this case the 25 independent variables, $X_{1k}$ = stands for the *k*th observation value for the independent variable $X_{1}$, and $\in_{k}$ is the error (disturbance) term that captures errors in model specification and other factors that influence healthcare workers’ EQ-VAS (overall health status score) but are not explicitly considered in the model.

The predictors of the healthcare workers’ overall health status were assessed using this model. A *t*-test was performed to determine whether each individual variable regression slope coefficient was statistically significant at 90% or 95% level of confidence.

### 2.5. Ethical Considerations

Following permission from the Meru County Government Department of health [CGM/COH/1/17(50)], permission was sought from all the hospitals that participated in this study. Subsequently, written informed consent was obtained from each respondent, before they anonymously and voluntarily completed the self-administered questionnaire. Before this, the research protocol underwent a sequential three-step approval process. In South Africa, the University of Pretoria, Faculty of Health Sciences Research Ethics Committee approved the protocol of this study [718/2019]. In Kenya, the United States International University Africa, Institutional Review Board, also granted Kenyan ethical approval [USIU-A/IRB/130-2020]. Subsequently, the National Commission for Science, Technology and Innovation, Kenya, granted a national research license number [901924] to perform this study in Kenya.

## 3. Results

The total number of respondents in this study was 553 healthcare workers out of 566. It yielded a response rate of 97.7% because thirteen questionnaires were excluded from data analysis, due to 50% or more questions not being answered. The response rate could be attributed to various factors, including the fact that the questionnaire was asking about the healthcare workers themselves, thus they were inclined to participate. As mentioned earlier, data collection was conducted during the country’s early onset of the COVID-19 pandemic, during which the healthcare workers’ workload was less because people generally avoided visiting hospitals, due to fear of contracting the contagious COVID-19 virus. No incentives were offered or given to respondents, they all voluntarily participated in this study.

### 3.1. Sample Characteristics

Table 1 presents the percentage frequency distributions of the personal and job-related characteristics of the healthcare workers, overall (*n* = 553), and by hospital ownership (sub-sample). From a total of 553 respondents, 74.48% worked in public hospitals and 21.52% in mission hospitals.

### 3.2. Work Environment Characteristics

Table 2 presents the frequency distributions and percentages of the work environment characteristics measured among the healthcare workers, in three categories overall (*n* = 553), public (*n* = 434) and, mission (*n* = 119) hospital ownerships.

### 3.3. EQ-5D-5L Health Profile, Index Value and EQ-VAS

The EQ-5D-5L health profile showed that approximately 66.55% of all the respondents reported no problems across all the five dimensions. Nevertheless, 33.45% of the healthcare workers in this study reported problems within the dimensions assessed. In public hospitals (*n* = 434), about 64.75% of the healthcare workers had no problems across the dimensions, but 35.25% experienced health problems across the dimensions. In mission hospitals (*n* = 119), 73.11% of the respondents did not experience any problems across the five dimensions, leaving 26.89% who confirmed experiencing health-related problems; thus implying that not all healthcare workers are at their best health state, with approximately more than 30% experiencing problems across all the dimensions assessed.

The median of the EQ-5D-5L index (IQR) value was 0.900 (0.595–0.900, on a scale of 0 to 1) overall and in both public and mission hospitals. This implies that the healthcare workers’ health profiles were relatively high, with a median score of 0.900, which was 0.1 below 1, where 1 signifies full health. However, there is room for improvement, because the EQ-5D-5L index value scores fell short of full health by a value of 0.1.

**Figure 1:**
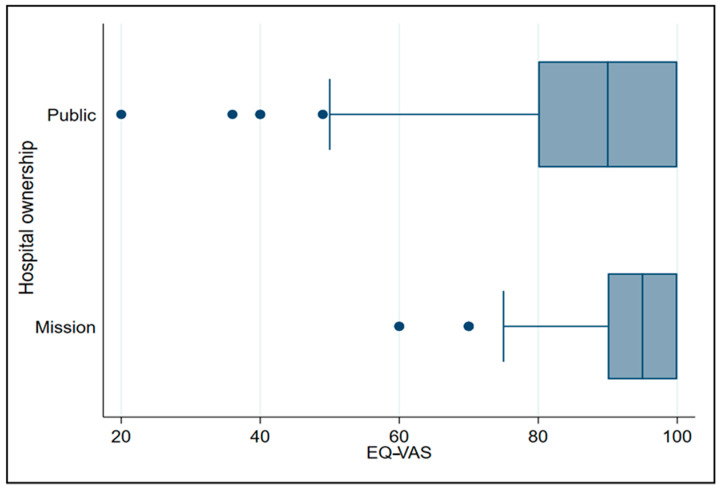
Box-and-whisker plot of EuroQol Visual Analogue Scale (EQ-VAS) by hospital ownership.

The EQ-VAS presented the results of the healthcare workers’ self-assessed overall health status, on a scale of 0–100 [43]. About 68.72% of the healthcare workers rated their overall health greater than 90 (where 100 indicates the best health you can imagine). The 553 respondents had a median of 90, first quartile = 80, third quartile = 100, minimum = 20 and maximum = 100, and four outliers = 20, 36, 40 and, 49. Figure 1 presents the box-and-whisker plots of EQ-VAS by hospital ownership.

Among the public hospitals (*n* = 434), the median (IQR) was 90 (80–100). Approximately 25% of the healthcare workers’ overall self-rated health status in public hospitals, was lower than 80. About 75% of the healthcare workers’ overall self-rated health status was rated more than 80, with four outliers. The healthcare workers in the mission hospitals (*n* = 119), the median of their overall self-rated health status was 95 (90–100). About 25% of their overall self-rated health status was rated lower than 90. Approximately 75% of their overall self-rated health status was rated more than 90, with two outliers.

### 3.4. Overall Self-Reported Health Status by Hospital Ownership

The ANOVA results revealed a statistically significant difference between the public and mission hospital healthcare workers’ overall self-reported health status (EQ-VAS) (*p* = 0.0057). Hospital ownership explained 1.38% of the variance in healthcare workers’ overall health status (see Table 3).

### 3.5. Predictors of Overall Health Status

The linear multivariate regression model showed that approximately 13.73% of the variance in the overall health status among the respondents was explained by the personal, job-related and, work environment characteristics (*p* < 0.01) (see Table 4).

Table 5 presents the results of the linear multivariate regression model, including the twenty-five independent variables assessed association with the EQ-VAS (overall self-rated health status). The model showed six statistically significant predictors of overall health status among the healthcare workers (*n* = 553). Hospital ownership (*p* = 0.029), age (*p* < 0.001) and income (*p* = 0.069) were the three significant personal and job-related predictors associated with the healthcare workers’ self-assessed health status. Moreover, the availability of water for handwashing (*p* = 0.018), presence of risk when using the toilet facilities (*p* = 0.015), and the overall safety of the hospital work environment (*p* = 0.001) were the three work environment-related predictors of healthcare workers’ overall health status (see Table 5). Twelve of the twenty-five independent variables had negative coefficients, which implies that as values of those independent variables increase, the healthcare workers’ overall health status decreases. On the other hand, thirteen variables had positive coefficients, meaning as the independent variables increase the overall health status of the healthcare workers increase.

## 4. Discussion

HRQOL is based on an individuals’ perception of their ability to execute functions associated with their health; related to the physical, psychological and occupational dimensions of life [24,43]. In this section, we discuss the HRQOL among healthcare workers in this study, compared to prior studies.

Overall, more than 30% of the healthcare workers studied reported experiencing problems across the dimensions. A study in South Africa found that up to 45% of the healthcare workers under study experienced problems across the five dimensions [28], thus the issue of HRQOL is in force. Both the South African study [28] and this study dispel the misconception that healthcare workers are automatically always in perfect health, due to their medical background. Thus, there is room for more action-oriented research to be done on healthcare workers’ HRQOL. Health managers should consider implementing programs on health promotion behavior, and self-efficacy, which have been reported to have a positive impact on HRQOL [45], and thus, could enhance the healthcare workers’ HRQOL.

In this study, a statistically significant difference between overall health status among healthcare workers in public and mission hospitals was revealed. As the hospital ownership changed from public to mission, the overall health status of healthcare workers increased by 3.079%. Healthcare workers in mission hospitals reported experiencing higher overall health status (73.11%) compared to their counterparts in public hospitals (64.75%). A study in Brazil revealed that healthcare workers in public hospitals had the lowest HRQOL scores compared to the private and philanthropic hospitals [25]. It appears that there is a need for interventions to increase HRQOL, especially in the public health sector. Most of the respondents in this study were from the public health sector, therefore policymakers and hospital managers should consider developing and implementing policy based on these research findings. Details regarding the predictors and possible solutions are discussed below.

Age was found to be a significant predictor of healthcare workers’ HRQOL in this study. As the age of the healthcare workers increased, their overall HRQOL decreased by 0.431%. However, a study in Brazil revealed the older health workers were, the better their HRQOL compared to their younger counterparts [29]. The differences between the results in Kenya and Brazil may be attributed to contextual or cultural differences which influence the perception of age. Based on these findings, age-friendly employment policies need to be developed and implemented within the hospitals and the health system at a large scale. Age-friendly employment policies, such as creating an ergonomic work environment supporting older healthcare workers, will enhance their health [46]. Guaranteed financial incentives, and relatively flexible work schedules that allow work–life balance are some age-friendly strategies that could promote older healthcare workers’ health and encourage them to work in the health system longer [46].

Income was positively and significantly associated with HRQOL among healthcare workers in this study. The higher the income of the healthcare workers under study, the higher their overall HRQOL. At an individual level, an association has been reported between income and health, particularly in situations where there are scarce goods and services available to the public [47]. In this context, this may partly explain the recurrent health worker strikes due to delayed or missed payments [13], which elicit feelings of scarcity and uncertainty of payment of the income for which they have worked and on which they greatly depend. Kenya’s age dependency ratio of 71.3% in 2019 indicates that children (0–14-year-olds) and the elderly (65 years and above) are dependent on those working for survival [48]. Therefore, delayed pay and missed pay among healthcare workers jeopardizes survival of health workers and their dependents, and aggravates the income inequalities which adversely affect population health [49] especially, in a lower-middle income such as Kenya with approximately 33.4% of the population living below the international poverty (Int$) line of Int$1.9 per day [50]. Thus, national and county policymakers should develop and implement strategies that facilitate timely payment and provide equal opportunities for promotions and incentives. This kind of action could potentially increase the HRQOL and eventually the retention of healthcare workers, especially in rural and remote areas in Kenya.

Previous studies revealed contrary results regarding the personal and job-related characteristics among healthcare workers. For instance, sex was a significant factor among health professionals in Turkey, where males had a higher HRQOL compared to their female colleagues [51]. This study revealed that sex was inversely related to HRQOL but was a non-significant characteristic among the respondents. In this study, the health professional cadre was also non-significant. A Turkish study on the other hand, reported higher HRQOL scores among physicians and health technicians compared to nurses and midwives [51]. Similarly, in Italy, the professional role significantly impacted the HRQOL, where nurses reported lower HRQOL scores compared to doctors and occupational safety and health technologists [52]. Although the professional cadre was a non-significant predictor in our study, further research needs to be done country-wide, to ascertain if this is similar or different in other locations. In addition, more studies on HRQOL across health professional cadres will inform future directions of health development, specific to the professional cadre needs [51].

In this study, length of work experience was a non-significant characteristic of healthcare workers’ HRQOL; this was similar to a Turkish study [51]. On the contrary, in Italy, the longer a healthcare workers’ career, the lower their general health score [52]. The differences in results could be attributed to contextual factors such as culture, location, and the period of study. As much as length of work experience was a non-significant predictor of HRQOL, age was a significant predictor in this study. The healthier healthcare workers are, the longer they are likely to work [46]. Hence, diversification of hospital organizing services, policies, and strategies such as age-friendly benefit packages promoting their health for example assistive devices were necessary: for example, comprehensive health insurance covers that facilitate restorative surgery, and acquisition of nutritional supplements [53], are some ways that could promote their health and, enable them work longer in the health system. However, the authors recognize that more research needs to be done in multiple settings to inform evidence-based policy and strategies towards promoting healthcare workers’ HRQOL for longer job retention.

A healthy and safe work environment is valued by health providers and is paramount to the health worker performance and retention [54]. Improved performance among healthcare workers has been attributed to safety and hygiene; this subsequently has increased client satisfaction [54]. In this study, as the overall safety of the hospital work environment increased, the overall HRQOL of the healthcare workers increased. The presence of risk when using the toilet facility decreased the overall HRQOL among the respondents. This finding implies that the higher the perceived risk of the hospital work environment, the lower the healthcare workers’ perception of their HRQOL. The healthcare workers’ availability of water for handwashing increased the overall health status by 4.478%. Weinberg and colleagues [55] reported that the high-performance work environment in hospitals significantly correlated with better performance, better retention, and better-quality healthcare among the healthcare providers. Thus, policymakers and hospital managers need to consider the benefits and importance of designing a high-performance work environment because of its potential benefits related to the quality of healthcare delivery and patient outcomes [55].

According to Herzberg’s Two Factor Theory on job attitudes [56], the predictors of healthcare workers’ HRQOL are income or salary and work environment. Following this theory, hygiene factors are also known as job dissatisfiers. Hygiene factors are extrinsic to the job [57]. In this study, low salary and poor work environment were major dissatisfiers. Thus, hospital managers and health authorities should be explicit in the implementation policies of salary increment, financial incentives and payment of healthcare workers [57]. In relation to the environment, hospital managers and health policymakers should eliminate the dissatisfaction contributing to a poor working environment. Based on the findings, this could be achieved through improving the hospital safety, hygiene and work environment, in order to make the work environment in hospitals satisfying for healthcare workers to have a better HRQOL and to perform optimally.

The healthier healthcare workers are, the better the relationship they will have with colleagues, and they will deliver better healthcare services to patients they encounter daily [52]. The results in this study should be viewed with some limitations in mind; hence, opportunities for future research.

Firstly, this was a cross-sectional study; therefore, only correlations could be reported. Future studies using a longitudinal approach to monitor and evaluate the HRQOL of healthcare workers are essential to capture the trends accurately and modify health policy accordingly. The second limitation is that the sample reflects the healthcare workers in one of the forty-seven counties in Kenya, thus limiting the generalizability of these results to the entire country. Future studies need to be done in the other 46 counties in order to assess the similarities and variations in HRQOL among the healthcare workers in the different localities countrywide. Thirdly, due to the self-reported nature of the questionnaire used, the possibility of response bias is present. To reduce the likelihood for such a bias, respondents were informed that the research was anonymous, and their honesty would be valued. In future research, the HRQOL could be assessed alongside government and mission programs aimed at improving the health and wellbeing of the health workforce.

## 5. Conclusions

This study highlighted personal, job-related, and work environment predictors of HRQOL among healthcare workers in public and mission hospitals Meru County, Kenya. It is evident that some personal, job-related, and work environment characteristics are significant predictors of HRQOL among healthcare workers. The majority of the respondents reported perfect health, thus through evidence-based policy development and implementation of HRQOL programs, other health workers with problems stand a chance of attaining a higher HRQOL. This study emphasizes the importance of involving the healthcare workers in the decision-making process of promoting their HRQOL, because some of our results differed with prior studies also among healthcare workers. It is evident that not every healthcare worker is in perfect health, as is the misconception based on their medical background. This finding implies that health policymakers and managers should aim at empowering and enhancing the changeable HRQOL among healthcare workers at the individual, organizational, and health system levels. Designing evidence-based medium- and long-term policies and programs would ensure effective implementation, and health workforce strengthening. In order to ensure sustainability within the national and county health systems, an inter-sectoral collaboration between the public and private sectors is recommended during the development (and revision) of health workforce policy aimed at HRQOL and wellbeing among healthcare workers in Kenya.

## Figures and Tables

**Table 1:** Overall and sub-sample percentage frequency distributions of personal and job-related characteristics.

Personal and Job-Related Variables	Overall (*n* = 553) *n* (%)	Public Hospitals (*n* = 434) *n* (%)	Mission Hospitals (*n* = 119) *n* (%)
Sex			
Male	214 (38.70)	180 (41.47)	34 (28.57)
Female	339 (61.30)	254 (58.53)	85 (71.43)
Age			
≤25	62 (11.21)	52 (11.98)	10 (8.40)
26–35	220 (39.78)	177 (40.78)	43 (36.13)
36–45	181(32.73)	134 (30.88)	47 (39.50)
46–55	67 (12.12)	57 (13.13)	10 (8.40)
≥56	23 (3.80)	14(3.23)	9 (7.56)
Marital status			
Single	179 (32.37)	140 (32.26)	39 (32.77)
Married	349 (63.11)	275 (62.67)	74 (62.18)
Divorced	12 (2.17)	8 (1.84)	4 (3.36)
Widowed	13 (2.35)	11 (2.53)	2 (1.68)
Years of experience			
<5	146 (26.40)	113 (26.04)	44 (36.97)
5–10	197 (35.62)	157 (36.18)	29 (24.37)
11–20	137 (24.77)	103 (23.73)	34 (28.57)
21–30	61 (11.03)	52 (11.98)	9 (7.56)
>30	12 (2.17)	9 (2.07)	3 (2.52)
Income range per month in Kenyan Shilling (KES)			
≤14,999	25 (4.52)	23 (5.30)	2 (1.68)
15,000–24,999	56 (10.13)	40 (9.22)	16 (13.45)
25,000–44,999	65 (11.75)	48 (11.06)	17 (14.29)
45,000–64,999	119 (21.52)	90 (20.74)	29 (24.37)
65,000–74,999	65 (11.75)	50 (11.52)	15 (12.61)
75,000–84,999	62 (11.21)	45 (10.37)	17 (14.29)
85,000–104,999	83 (15.01)	73 (16.82)	10 (8.40)
≥105,000	78 (14.10)	65 (14.97)	13 (10.92)
Education attained			
Certificate	28 (5.06)	20 (4.61)	8 (6.72)
Diploma	335 (60.58)	260 (59.91)	75 (63.03)
Bachelor’s degree	157 (28.39)	128 (29.49)	29 (24.37)
Honors degree	1 (0.18)	1 (0.23)	0 (0)
Master’s degree	30 (5.42)	24 (5.53)	6 (5.04)
Doctor of Philosophy (PhD) degree	2 (0.36)	1 (0.23)	1 (0.84)
Health professional cadres			
Physician or Specialist Doctor	31 (5.61)	18 (4.15)	13 (10.92)
Nursing professional	169 (30.56)	134 (30.88)	35 (29.41)
Pharmaceutical professional	41 (7.41)	30 (6.91)	11 (9.24)
Dentistry professional	52 (9.40)	37 (8.53)	15 (12.61)
Clinical officer	100 (18.08)	76 (17.51)	24 (20.17)
Medical laboratory scientist	54 (9.76)	41 (9.45)	13 (10.92)
Public health specialist	40 (7.23)	37 (8.53)	3 (2.52)
Nutrition and dietetics	17 (3.07)	16 (3.69)	1 (0.84)
Radiographer	12 (2.17)	10 (2.30)	2 (1.68)
Health records officer	20 (3.62)	20 (4.61)	0 (0)
Physiotherapist	12 (2.17)	10 (2.30)	2 (1.68)
Mental health specialists	5 (0.90)	5 (1.15)	0 (0)
Type of employment			
Full-time	517 (93.49)	406 (93.55)	111 (93.28)
Part-time	36 (6.51)	28 (6.45)	8 (6.72)
Hours worked per week			
≤10	47 (8.50)	42 (9.68)	5 (4.20)
11–20	5 (0.90)	4 (0.92)	1 (0.84)
21–30	11 (1.99)	10 (2.30)	1 (0.84)
31–40	407 (73.60)	316 (72.81)	91 (76.47)
41–50	45 (8.14)	34 (7.83)	11 (9.24)
≥51	38 (6.87)	28 (6.45)	10 (8.40)
Household size			
1–2	181 (32.73)	127 (29.26)	54 (45.38)
3–4	200 (36.17)	161 (37.10)	39 (32.77)
5–7	159 (28.75)	133 (30.65)	26 (21.85)
8–0	13 (2.35)	13 (3.0)	0 (0)
Upgrade in-service training			
Yes	365 (66)	283 (65.21)	82 (68.91)
No	188 (34)	151 (34.79)	37 (31.09)
Staff housing			
Yes	74 (13.38)	39 (8.99)	35 (29.41)
No	479 (86.62)	395 (91.01)	84 (70.59)
Type of housing			
Permanent housing	64 (11.57)	35 (8.06)	29 (24.37)
Semi-permanent housing	9 (1.63)	3 (0.69)	6 (5.04)
Temporary housing	1 (0.18)	1 (0.23)	1 (0.84)

Note: The exchange rate as of 22 December 2020, was, USD 1 = KES 110.38.

**Table 2:** Overall and sub-sample percentage frequency distribution of work environment characteristics (*n* = 553).

Work Environment Variables	Overall (*n* = 553) *n* (%)	Public Hospitals (*n* = 434) *n* (%)	Mission Hospitals (*n* = 119) *n* (%)
Constant supply of water			
Yes	459 (83)	353 (81.33)	106 (89.08)
No	94 (17)	81 (18.66)	13 (10.92)
Occurrence of unavailable water (≥1 day)			
Yes	220 (39.78)	161 (37.10)	59 (49.58)
No	333 (60.22)	273 (62.90)	60 (50.42)
Safe drinking water			
Yes	413 (74.68)	310 (71.43)	103 (86.55)
No	140 (25.32)	124 (28.57)	16 (13.45)
Acceptable main source of water			
Yes	461 (83.36)	353 (81.34)	108 (90.76)
No	92 (16.64)	81 (18.66)	11 (9.24)
Type of toilet facility			
Flush or pour flush	420 (75.95)	323 (74.42)	97 (81.51)
Pit latrine	133 (24.05)	111 (25.58)	22 (18.49)
Risk when using toilet facility			
Yes	141 (25.50)	120 (27.65)	21 (17.65)
No	412 (74.50)	314 (72.35)	98 (82.35)
Type of risk			
Injury	16 (2.89)	15 (3.46)	1 (0.84)
Harassment	15 (2.71)	15 (3.46)	0 (0)
Health (infections)	99 (17.90)	80 (18.43)	19 (15.97)
≥2 types of risk	11 (1.99)	11 (2.53)	0 (0)
None	412 (74.50)	313 (72.12)	99 (83.19)
Hospital disposal of waste			
Formal collection service	100 (18.08)	75 (17.28)	25 (21.01)
Informal collection service	15 (2.71)	9 (2.07)	6 (5.04)
Disposal in designated area	181 (32.73)	144 (33.18)	37 (31.09)
Disposal within the hospital compound	138 (24.95)	102 (23.50)	36 (30.25)
Disposal elsewhere (burning, burying or other)	52 (9.40)	45 (10.37)	7 (5.88)
Unknown	67 (12.12)	59 (13.59)	8 (6.72)
Constant availability of handwash soap			
Yes	508 (91.86)	396 (91.24)	112 (94.11)
No	45 (8.14)	38 (8.76)	7 (5.88)
Constant availability of water for handwashing			
Yes	468 (84.63)	361 (83.18)	107 (89.92)
No	85 (15.37)	73 (16.82)	12 (10.08)
Appropriate distance of handwashing station from the toilet (≤5 m)			
Yes	499 (90.24)	389 (89.63)	110 (92.44)
No	54 (9.76)	45 (10.37)	9 (7.56)
Workplace safety and health committee			
Yes	313 (56.60)	262 (60.37)	51 (42.86)
No	240 (43.40)	172 (39.63)	68 (57.14)
Overall safety of hospital work environment			
0–2 (not safe)	11 (1.99)	11 (2.53)	0 (0)
3–5 (slightly safe)	99 (17.90)	90 (20.74)	9 (7.56)
6–8 (moderately safe)	300 (54.25)	229 (52.76)	71 (59.66)
9–10 (very safe)	143 (5.86)	104 (23.96)	39 (32.77)

**Table 3:** ANOVA of EQ-VAS and hospital ownership.

	*n*	R^2^	Adjusted R^2^	F	Sig. F Change
EQ-VAS	553	0.0138	0.0120	7.69	0.0057 *

* *p* < 0.05 indicates statistical significance.

**Table 4:** Linear multivariate regression model results.

	*n*	R^2^	Adjusted R^2^	F	Sig. F Change
EQ-VAS	553	0.1373	0.964	3.35	0.0001 *

* *p* < 0.01 indicates statistical significance.

**Table 5:** Results of linear multivariate regression of overall health status, and independent variables (*n* = 553).

Independent Variables	Coef.	Std. Err.	T-Value	Sig.	95% Conf. Interval	
Hospital Ownership	3.079	1.407	2.19	0.029 *	0.316	5.843
Sex	−0.072	1.093	−0.07	0.947	−2.220	2.075
Age	−0.431	0.103	−4.18	0.000 *	−0.634	−0.229
Income	2.550	1.401	1.82	0.069 **	−0.202	5.303
Marital status	1.329	1.245	1.07	0.286	−1.117	3.775
Education attained	0.006	1.170	0.01	0.996	−2.293	2.305
Years of experience	0.181	0.120	1.51	0.132	−0.055	0.416
Health professional cadre	0.435	1.208	0.36	0.719	−1.937	2.807
Type of employment	−0.599	2.226	−0.27	0.788	−4.974	3.773
In-service training	−0.440	1.180	−0.37	0.709	−2.757	1.877
Hours worked per week	0.021	0.039	0.53	0.595	−0.056	0.098
Household size	0.065	0.328	0.20	0.843	−0.579	0.708
Staff housing	−1.703	1.612	−1.06	0.291	−4.871	1.464
Consistent supply of water	0.602	1.698	0.35	0.723	−2.733	3.938
Occurrence of water unavailability	−0.956	1.148	−0.83	0.405	−3.211	1.299
Safe drinking water	−0.177	1.407	−0.13	0.900	−2.941	2.587
Acceptable main source of water	−1.020	1.675	−0.61	0.543	−4.310	2.670
Type of toilet facility	−0.951	1.265	−0.75	0.453	−3.437	1.535
Presence of risk when using toilet facility	−3.126	1.287	−2.43	0.015 *	−5.654	−0.597
Hospital dispose of garbage	0.038	1.373	0.03	0.978	−2.660	2.736
Availability of water for hand washing	4.478	1.895	2.36	0.018 *	0.756	8.200
Constant availability of soap	2.284	2.258	1.01	0.312	−2.153	6.721
≤5 m of handwashing station from the toilet	−1.767	2.112	−0.84	0.403	−5.917	2.382
Workplace safety and health committee	−1.238	1.211	−1.02	0.307	−3.617	1.141
Overall safety of hospital work environment	1.030	0.309	3.34	0.001 *	0.423	1.637
Constant	90.257	4.630	19.49	0.000	81.160	99.354

* *p* < 0.01 indicates statistical significance at 95% confidence level; ** *p* < 0.01 indicates statistical significance at 90% confidence level.

## Data Availability

The data presented in this study are available on request from the corresponding author. The data are not publicly available until this article is published.
